# Supplementary material for: Neuroplasticity within and between Functional Brain Networks in Mental Training Based on Long-Term Meditation
Source: Brain Sci. 2021 Aug 18;11(8):1086. doi: 10.3390/brainsci11081086 (PMC8393942; doi:10.3390/brainsci11081086)
Supplement: Supplementary file 1 [file brainsci-11-01086-s001.zip › brainsci-1317953-supplementary.pdf]

Supplementary Information

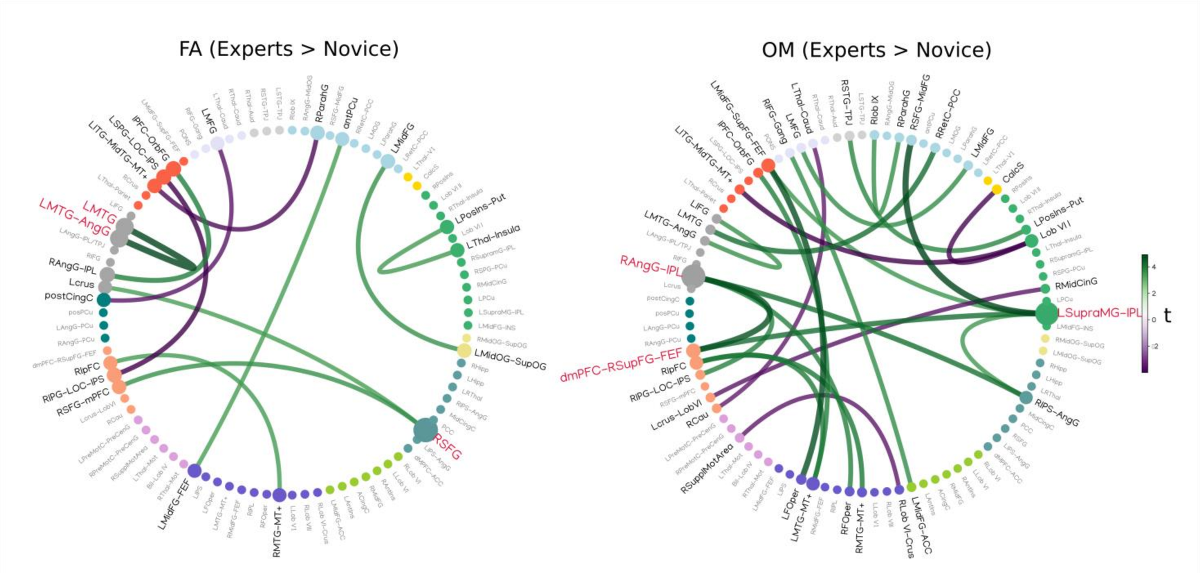

Figure S1: *Differential connectivities between meditators and control group.* Figure shows the connectivities that are statistically different in experts > controls (two-sided t-test; p<0.01 FDR corrected), in FA (left panel) and OM (right panel).

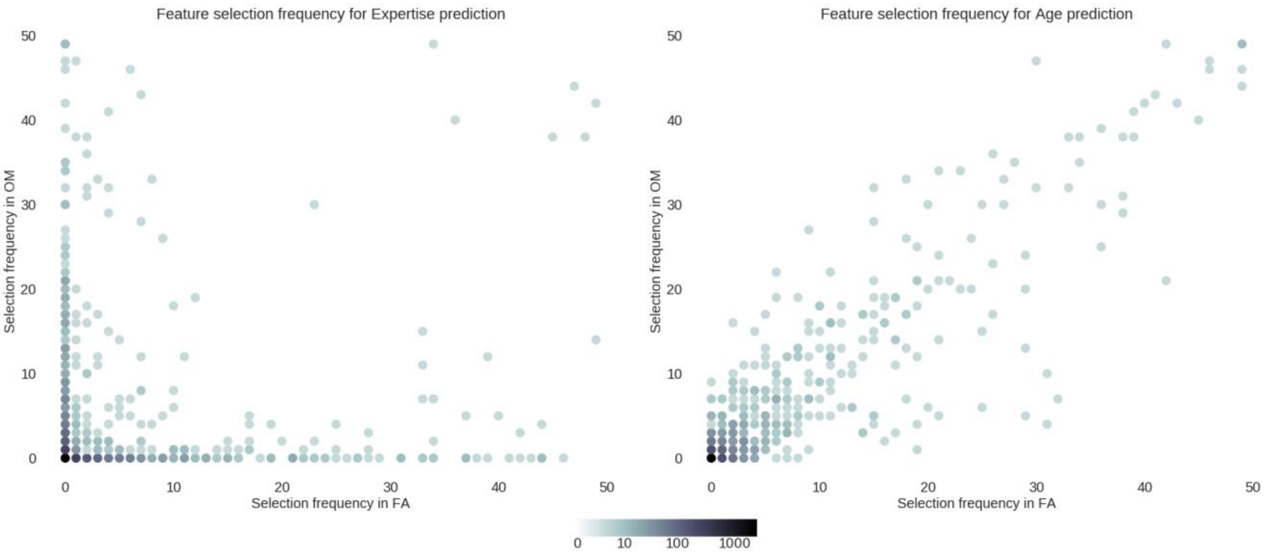

Figure S2: *Feature selection frequency for expertise and age prediction in control analysis.* Figure shows feature selection frequency for meditation expertise prediction (panel a) and age prediction (panel b), for focused attention (x-axis) and open monitoring meditation styles (y-axis). The maximum frequency value is equal to the number of cross-validation folds. The color of the dots indicates the number of features with that particular selection frequency.

Table S1: List of selected ROIs. Coordinates (x,y,z) of ROIs used for functional connectivity analysis, according to MNI atlas.

Coordinates			Regions	Labels	Network	Voxels
-56	-14	7	Left Superior Temporal/ Heschl's Gyrus	LSTG-TPJ	Auditory	962
56	-7	6	Right Superior Temporal Gyrus	RSTG-TPJ	Auditory	554
13	-16	-2	Right Thalamus	RThal-Aud	Auditory	26
14	-2	8	Right Thalamus. Caudate	RThal-Caud	Basal Ganglia	669
-14	-3	7	Left Thalamus. Caudate.	LThal-Caud	Basal Ganglia	828
-44	22	23	Left Inferior Frontal Gyrus	LMFG	Basal Ganglia	18
48	28	17	Right Inferior Frontal Gyrus	RIFG-Gang	Basal Ganglia	63
-6	-26	-38	Pons	PONS	Basal Ganglia	32
-30	23	49	Left Middle Frontal eye Fields.	LMidFG-SupFG-FEF	LECN	1501
-40	48	-1	Left Inferior / Orbito Frontal Gyrus	lPFC-OrbFG	LECN	437
-42	-63	46	Left Superior / Inferior Parietal Sulcus.	LSPG-LOC-IPS	LECN	2110
-59	-42	-12	Left Inferior / Middle Temporal Gyrus	LITG-MidTG-MT+	LECN	350
36	-69	-43	Right Crus I	RCrus	LECN	310
-14	-28	2	Left Thalamus	LThal-Pariet	LECN	8
-49	25	-4	Inferior Frontal Gyrus	LIFG	Language	652
-52	-1	-22	Left Middle Temporal Gyrus	LMTG	Language	27
-52	-31	-6	Left Middle Temporal Gyrus. Angular Gyrus	LMTG-AngG	Language	317
-54	-55	22	Left Angular Gyrus. Inferior Parietal Lobule.	LAngG-IPL/TPJ	Language	1420
49	28	-10	Right Inferior Frontal Gyrus	RIFG	Language	58
55	-43	10	Right Angular Gyrus. Inferior Parietal Lobule.	RAngG-IPL	Language	1106
-22	-80	-35	Left Crus I	LCrus	Language	270
2	-28	27	Midcingulate / Posterior Cingulate Cortex	postCingC	Precuneus	579
3	-72	40	Posterio Precuneus	posPCu	Precuneus	1572
-36	-62	46	Left Angular Gyrus	LAngG-PCu	Precuneus	388
39	-62	45	Right Angular Gyrus	RAngG-PCu	Precuneus	96
38	26	42	Dorsomedial Prefrontal Cortex.	dmPFC-RSupFG-FEF	RECN	2093
38	54	1	Right Left Posterior Frontal Cortex	RlpFC	RECN	356
48	-54	47	Right Inferior Parietal Gyrus. Intraparietal Sulcus	RIPG-LOC-IPS	RECN	1873
5	37	46	Right Superior Frontal Gyrus	RSFG-mPFC	RECN	83
-30	-73	-39	Left Crus 1. Crus II. Lobule VI	Lcrus-LobVI	RECN	2403
13	2	14	Right Caudate	Rcau	RECN	188
-33	-21	60	Left Precentral / Postcentral Gyrus	LPreMotC-PreCenG	Sensorimotor	1365
38	-18	57	Right Precentral / Postcentral Gyrus	RPreMotC-PreCenG	Sensorimotor	1446
3	-13	61	Right Supplementary Motor Area	RSupplMotArea	Sensorimotor	159
-12	-19	-1	Left Thalamus	LThal-Mot	Sensorimotor	19
-2	-52	-19	Bilateral Lobule IV	Bil-Lob IV	Sensorimotor	2015
12	-20	-4	Right Thalamus	RThal-Mot	Sensorimotor	20
-27	-1	54	Left Middle Frontal Gyrus. Frontal Eye Fields	LMidFG-FEF	Visuospatial	338
-36	-46	47	Left Intraparietal Sulcus	LIPS	Visuospatial	2020
-47	13	27	Left Frontal Operculum. Inferior Frontal Gyrus	LFOper	Visuospatial	1105
-49	-65	-6	Left Middle Temporal Gyrus	LMTG-MT+	Visuospatial	93
28	2	54	Right Middle Frontal Gyrus	RMidFG-FEF	Visuospatial	97
37	-47	48	Right Inferior Parietal Lobule	RIPL	Visuospatial	1193
49	12	28	Right Frontal Operculum.	RFOper	Visuospatial	326
50	-59	-11	Right Middle Temporal Gyrus	RMTG-MT+	Visuospatial	76
-27	-72	-52	Left Lobule V111.	LLob V1-FEF	Visuospatial	38
24	-73	-51	Right Lobule VIII.	RLob VIII	Visuospatial	131
34	-70	-26	Right Lobule VI. Crus I	RLob VI-Crus	Visuospatial	62

-31	47	22	Left Middle Frontal Gyrus	LMidFG-ACC	anterior Salience	651
-42	14	-3	Left Anterior Insula	LAntIns	anterior Salience	305
0	17	47	Anterior Cingulate Cortex.	ACingC	anterior Salience	2887
28	46	26	Right Middle Frontal Gyrus	RMidFG	anterior Salience	470
43	15	-1	Right Anterior Insula	RAntIns	anterior Salience	319
-34	-56	-32	Left Lobule VI. Crus I	LLob VI-Insula	anterior Salience	95
36	-58	-32	Right Lobule VI. Crus I	RLob VI	anterior Salience	139
-3	49	14	Medial Prefrontal / Anterior Cingulate Cortex.	dMPFC-ACC	dorsal DMN	5257
-48	-68	35	Left Intraparietal Sulcus. Angular Gyrus	LIPS-AngG	dorsal DMN	97
19	38	47	Right Superior Frontal Gyrus	RSFG	dorsal DMN	137
1	-53	28	Posterior Cingulate Cortex. Precuneus	PCC	dorsal DMN	1555
2	-15	36	Midcingulate Cortex	MidCingC	dorsal DMN	114
50	-64	32	Right Intraparietal Sulcus. Angular Gyrus	RIPS-AngG	dorsal DMN	38
-1	-8	4	Left and Right Thalamus	LRThal	dorsal DMN	220
-24	-29	-13	Left Hippocampus	LHipp	dorsal DMN	393
27	-23	-17	Right Hippocampus	RHipp	dorsal DMN	142
-29	-87	-1	Left Middle / Superior Occipital Gyrus	LMidOG-SupOG	high Visual	868
32	-85	0	Right Middle /Superior Occipital Gyrus	RMidOG-SupOG	high Visual	1679
-39	35	30	Left Middle Frontal Gyrus	LMidFG-INS	posterior Salience	93
-57	-38	37	Left Supramarginal/ Inferior Parietal Gyrus	LSupraMG-IPL	posterior Salience	1205
-8	-52	61	Left Precuneus	LPCu	posterior Salience	98
12	-28	45	Right Midcingulate Cortex	RMidCinG	posterior Salience	56
21	-48	69	Right Superior Parietal Gyrus. Precuneus	RSPG-PCu	posterior Salience	133
59	-32	36	Right Supramarginal/ Inferior Parietal Gyrus	RSupramG-IPL	posterior Salience	1002
-12	-21	5	Left Thalamus	LThal-Insula	posterior Salience	142
-33	-41	-37	Lobule VI I	Lob VI I	posterior Salience	102
-37	-13	-5	Left Posterior Insula. Putamen	LPosIns-Put	posterior Salience	114
13	-14	9	Right Thalamus	RThal-Insula	posterior Salience	63
36	-43	-40	Lobule VI II	Lob VI II	posterior Salience	13
40	-6	-9	Right Posterior Insula	RPosIns	posterior Salience	134
0	-74	11	Calcarine Sulcus	CalcS	primary Visual	1116
-18	-24	-4	Left Thalamus	LThal-V1	primary Visual	4
-12	-58	15	Left Retrosplenial and Posterior Cingulate Cortex	LRetC-PCC	ventral DMN	462
-24	12	55	Left Middle Frontal Gyrus	LMidFG	ventral DMN	405
-28	-37	-15	Left Parahippocampal Gyrus	LParahG	ventral DMN	134
-36	-81	32	Left Middle Occipital Gyrus	LMOG	ventral DMN	491
13	-53	14	Right Retrosplenial and Posterior Cingulate Cortex	RRetC-PCC	ventral DMN	590
1	-57	54	Anterior Precuneus	antPCu	ventral DMN	1921
26	26	45	Right Superior / Middle Frontal Gyrus	RSFG-MidFG	ventral DMN	399
28	-33	-19	Right Parahippocampal Gyrus	RParahG	ventral DMN	90
43	-74	32	Right Angular /Middle Occipital Gyrus	RAngG-MidOG	ventral DMN	752
15	-46	-53	Right Lobule IX	Rlob IX	ventral DMN	81
